# Stem cell therapy: a potential approach for treatment of influenza virus and coronavirus-induced acute lung injury

**DOI:** 10.1186/s13287-020-01699-3

**Published:** 2020-05-24

**Authors:** Jiang Du, Han Li, Jie Lian, Xinxing Zhu, Liang Qiao, Juntang Lin

**Affiliations:** 1 College of Biomedical Engineering, Xinxiang Medical University, Xinxiang, 453003 China; 2 Stem Cell and Biotherapy Engineering Research Center of Henan, Xinxiang Medical University, East of JinSui Road #601, Xinxiang City, Xinxiang, 453003 Henan Province China; 3 College of Life Science and Technology, Xinxiang Medical University, Xinxiang, 453003 China

**Keywords:** Stem cell therapy, Acute lung injury, Influenza virus, Coronaviruses, Mesenchymal stem cells, Lung stem/progenitor cells

## Abstract

Acute lung injury (ALI), an increasingly devastating human disorder, is characterized by a multitude of lung changes arising from a wide variety of lung injuries. Viral infection is the main cause of morbidity and mortality in ALI and acute respiratory distress syndrome (ARDS) patients. In particular, influenza virus, coronavirus, and other respiratory viruses circulate in nature in various animal species and can cause severe and rapidly spread human infections. Although scientific advancements have allowed for rapid progress to be made to understand the pathogenesis and develop therapeutics after each viral pandemic, few effective methods to treat virus-induced ALI have been described. Recently, stem cell therapy has been widely used in the treatment of various diseases, including ALI. In this review, we detail the present stem cell-based therapeutics for lung injury caused by influenza virus and the outlook for the future state of stem cell therapy to deal with emerging influenza and coronaviruses.

## Background

Acute lung injury (ALI) is a devastating disease process involving pulmonary edema and atelectasis caused by capillary membrane injury [1]. The main clinical manifestation is the acute onset of hypoxic respiratory failure, which can subsequently trigger a cascade of serious complications and even death [2]. Thus, ALI causes a considerable financial burden for health care systems throughout the world. ALI can result from various causes, including multiple traumas, large-volume blood transfusions, and bacterial and viral infections [2, 3]. A variety of viruses, including influenza virus, coronavirus (CoV), adenovirus, cytomegalovirus (CMV), and respiratory syncytial virus (RSV), are associated with ALI [4]. Importantly, most viruses, whose hosts are various animal species, can cause severe and rapidly spreading human infections. In the early 2000s, several outbreaks of influenza virus and CoV emerged, causing human respiratory and intestinal diseases worldwide, including the more recent SARS-CoV-2 infection [5–7]. To date, SARS-CoV-2 has affected more than 80,000 people, causing nearly 3300 deaths in China and more than 1,800,000 people, causing nearly 110,000 deaths all over the world (http://2019ncov.chinacdc.cn/2019-nCoV/).

Infectious respiratory diseases caused by different viruses are associated with similar respiratory symptoms ranging from the common cold to severe acute respiratory syndrome [8]. This makes the clinical distinction between different agents involved in infection very difficult [8, 9]. Currently, the clinical experience mainly includes antibacterial and antiviral drug treatment derived from handling several outbreaks of influenza virus and human CoVs. Numerous agents have been identified to inhibit the entry and/or replication of these viruses in cell culture or animal models [10]. Although these antiviral drugs can effectively prevent and eliminate the virus, the full recovery from pneumonia and ALI depends on the resistance of the patient. Recently, stem cell-based therapy has become a potential approved tool for the treatment of virus-induced lung injury [11–13]. Here, we will give a brief overview of influenza virus and CoVs and then present the cell-based therapeutic options for lung injury caused by different kinds of viruses.

### Overview of influenza virus and human CoVs

Influenza virus and human CoV are the two most threatening viruses for infectious lung injury [14]. These pathogens can be transmitted through direct or indirect physical contact, droplets, or aerosols, with increasing evidence suggesting that airborne transmission, including via droplets or aerosols, enhances the efficiency of viral transmission among humans and causes uncontrolled infectious disease [15]. Throughout human history, outbreaks and occasional pandemics caused by influenza virus and CoV have led to approximately hundreds of millions of deaths worldwide [16].

Influenza virus is a well-known human pathogen that has a negative-sense RNA genome [17]. According to its distinct antigenic properties, the influenza virus can be divided into 4 subtypes, types A, B, C, and D. Influenza A virus (IAV) lineages in animal populations cause economically important respiratory disease. Little is known about the other human influenza virus types B, C, and D [18]. Further subtypes are characterized by the genetic and antigenic properties of the hemagglutinin (HA) and neuraminidase (NA) glycoproteins [19]. Sporadic and seasonal infections in swine with avian influenza viruses of various subtypes have been reported. The most recent human pandemic viruses—H1N1 from swine and H5N1 from avian—cause severe respiratory tract disease and lung injury in humans [20, 21].

CoVs, a large family of single-stranded RNA viruses, typically affect the respiratory tract of mammals, including humans. CoVs are further divided into four genera: alpha-, beta-, gamma-, and delta-CoVs. Alpha- and beta-CoVs can infect mammals, and gamma- and delta-CoVs tend to infect birds, but some of these viruses can also be transmitted to mammals [22]. Human CoVs were considered relatively harmless respiratory pathogens in the past. Infections with the human CoV strains 229E, OC43, NL63, and HKU1 usually result in mild respiratory illness, such as the common cold [23]. In contrast, the CoV responsible for the 2002 severe acute respiratory syndrome (SARS-CoV), the 2012 Middle East respiratory syndrome CoV (MERS-CoV), and, more recently, the SARS-CoV-2 have received global attention owing to their genetic variation and rapid spread in human populations [5–7].

Usually, the influenza virus can enter the columnar epithelial cells of the respiratory tract, such as the trachea, bronchi, and bronchioles. Subsequently, the influenza virus begins to replicate for an asymptomatic period of time and then migrate to the lung tissue to cause acute lung and respiratory injury [24]. Similar to those with influenza virus infection, patients with SARS, MERS, or SARS-CoV-2 present with various clinical features, ranging from asymptomatic or mild respiratory illness to severe ALI, even with multiple organ failure [5–7]. The pathogenesis of ALI caused by influenza virus and human CoV is often associated with rapid viral replication, marked inflammatory cell infiltration, and elevated proinflammatory cytokine/chemokine responses [25]. Interestingly, in IAV- and human CoV-infected individuals, the pulmonary pathology always involves diffuse alveolar damage, but viral RNA is present in only a subset of patients [26]. Some studies suggest that an overly exaggerated immune response, rather than uncontrolled viral spread, is the primary cause in fatal cases caused by virus infection [27]. Several immune cell types have been found to contribute to damaging host responses, providing novel approaches for therapeutic intervention [28].

### Stem cell therapy for influenza virus-induced lung injury

IAV infection, the most common cause of viral pneumonia, causes substantial seasonal and pandemic morbidity and mortality [29]. Currently, antiviral drugs are the primary treatment strategy for influenza-induced pneumonia. However, antiviral drugs cannot repair damaged lung cells. Here, we summarize the present studies of stem cell therapy for influenza virus-induced lung injury.

Mesenchymal stem/stromal cells (MSCs) constitute a heterogeneous subset of stromal regenerative cells that can be harvested from several adult tissue types, including bone marrow, umbilical cord, adipose, and endometrium [30]. They retain the expression of the markers CD29, CD73, CD90, and CD105 and have a rapid proliferation rate, low immunogenicity, and low tumorigenicity [30]. MSCs also have self-renewal and multidifferentiation capabilities and exert immunomodulatory and tissue repair effects by secreting trophic factors, cytokines, and chemokines [31]. Due to these beneficial biological properties, MSCs and their derivatives are attractive as cellular therapies for various inflammatory diseases, including virus-induced lung injury.

Several studies on IAV-infected animal models have shown the beneficial effects of the administration of different tissue-derived MSCs [32–35]. H5N1 virus infection reduces alveolar fluid clearance (AFC) and enhances alveolar protein permeability (APP) in human alveolar epithelial cells, which can be inhibited by coculture with human bone marrow-derived MSCs (BMSCs) [32]. Mechanistically, this process can be mediated by human BMSC secreted angiopoietin-1 (Ang1) and keratinocyte growth factor (KGF) [32]. Moreover, in vivo experiments have shown that human BMSCs have a significant anti-inflammatory effect by increasing the number of M2 macrophages and releasing various cytokines and chemokines, such as interleukin (IL)-1β, IL-4, IL-6, IL-8, and IL-17 [32]. Similar anti-inflammatory effects have been achieved in another virus-induced lung injury model. The intravenous injection of mouse BMSCs into H9N2 virus-infected mice significantly attenuates H9N2 virus-induced pulmonary inflammation by reducing chemokine (GM-CSF, MCP-1, KC, MIP-1α, and MIG) and proinflammatory cytokine (IL-1α, IL-6, TNF-α, and IFN-γ) levels, as well as reducing inflammatory cell recruitment into the lungs [33]. Another study on human BMSCs cocultured with CD8+ T cells showed that MSCs inhibit the proliferation of virus-specific CD8+ T cells and the release of IFN-γ by specific CD8+ T cells [36].

In addition, human umbilical cord-derived MSCs (UC-MSCs) were found to have a similar effect as BMSCs on AFC, APP, and inflammation by secreting growth factors, including Ang1 and hepatocyte growth factor (HGF), in an in vitro lung injury model induced by H5N1 virus [34]. UC-MSCs also promote lung injury mouse survival, increase the body weight, and decreased the APP levels and inflammation in vivo [34]. Unlike Ang1, KGF, and HGF mentioned above, basic fibroblast growth factor 2 (FGF2) plays an important role in lung injury therapy via immunoregulation. The administration of the recombinant FGF2 protein improves H1N1-induced mouse lung injury and promotes the survival of infected mice by recruiting and activating neutrophils via the FGFR2-PI3K-AKT-NFκB signaling pathway [37]. FGF2-overexpressing MSCs have an enhanced therapeutic effect on lipopolysaccharide-induced ALI, as assessed by the proinflammatory factor level, neutrophil quantity, and histopathological index of the lung [38].

MSCs secrete various soluble factors and extracellular vesicles (EVs), which carry lipids, proteins, DNA, mRNA, microRNAs, small RNAs, and organelles. These biologically active components can be transferred to recipient cells to exert anti-inflammatory, antiapoptotic, and tissue regeneration functions [39]. EVs isolated from conditioned medium of pig BMSCs have been demonstrated to have anti-apoptosis, anti-inflammation, and antiviral replication functions in H1N1-affected lung epithelial cells and alleviate H1N1-induced lung injury in pigs [35]. Moreover, the preincubation of EVs with RNase abrogates their anti-influenza activity, suggesting that the anti-influenza activity of EVs is due to the transfer of RNAs from EVs to epithelial cells [35]. Exosomes are a subset of EVs that are 50–200 nm in diameter and positive for CD63 and CD81 [40]. Exosomes isolated from the conditioned medium of UC-MSCs restore the impaired AFC and decreased APP in alveolar epithelial cells affected by H5N1 virus [34]. In addition, the ability of UC-MSCs to increase AFC is superior to that of exosomes, which indicates that other components secreted by UC-MSCs have synergistic effects with exosomes [34].

Despite accumulating evidence demonstrating the therapeutic effects of MSC administration in various preclinical models of lung injury, some studies have shown contrasting results. Darwish and colleagues proved that neither the prophylactic nor therapeutic administration of murine or human BMSCs could decrease pulmonary inflammation or prevent the progression of ALI in H1N1 virus-infected mice [41]. In addition, combining MSC administration with the antiviral agent oseltamivir was also found to be ineffective [41]. Similar negative results were obtained in another preclinical study. Murine or human BMSCs were administered intravenously to H1N1-induced ARDS mice [42]. Although murine BMSCs prevented influenza-induced thrombocytosis and caused a modest reduction in lung viral load, murine or human BMSCs failed to improve influenza-mediated lung injury as assessed by weight loss, the lung water content, and bronchoalveolar lavage inflammation and histology, which is consistent with Darwish’s findings [42]. However, the mild reduction in viral load observed in response to murine BMSC treatment suggests that, on balance, MSCs are mildly immunostimulatory in this model [42]. Although there are some controversial incidents in preclinical research, the transplant of menstrual-blood-derived MSCs into patients with H7N9-induced ARDS was conducted at a single center through an open-label clinical trial (http://www.chictr.org.cn/). MSC transplantation significantly lowered the mortality and did not result in harmful effects in the bodies of the patients [43]. This clinic study suggests that MSCs significantly improve the survival rate of influenza virus-induced lung injury.

The effects of exogenous MSCs are exerted through their isolation and injection into test animals. There are also some stem/progenitor cells that can be activated to proliferate when various tissues are injured. Basal cells (BCs), distributed throughout the pseudostratified epithelium from the trachea to the bronchioles, are a class of multipotent tissue-specific stem cells from various organs, including the skin, esophagus, and olfactory and airway epithelia [44, 45]. Previously, TPR63+/KRT5+ BCs were shown to self-renew and divide into club cells and ciliated cells to maintain the pseudostratified epithelium of proximal airways [46]. Several studies have shown that TPR63+/KRT5+ BCs play a key role in lung repair and regeneration after influenza virus infection. When animals typically recover from H1N1 influenza infection, TPR63+/KRT5+ BCs accumulate robustly in the lung parenchyma and initiate an injury repair process to maintain normal lung function by differentiating into mature epithelium [47]. Lineage-negative epithelial stem/progenitor (LNEP) cells, present in the normal distal lung, can activate a TPR63+/KRT5+ remodeling program through Notch signaling after H1N1 influenza infection [48]. Moreover, a population of SOX2+/SCGB1A−/KRT5− progenitor cells can generate nascent KRT5+ cells as an early response to airway injury upon H1N1 influenza virus infection [49]. In addition, a rare p63+Krt5− progenitor cell population also responds to H1N1 virus-induced severe injury [50]. This evidence suggests that these endogenous lung stem/progenitor cells (LSCs) play a critical role in the repopulation of damaged lung tissue following severe influenza virus infection (Table 2).

Taken together, the present in vitro (Table 1) and in vivo (Table 2) results show that MSCs and LSCs are potential cell sources to treat influenza virus-induced lung injury.

**Table 1:** MSCs treatment for influenza virus induced lung injury in vitro

Cell sources	Passage number	Influenza virus	Cell models	Biological effect
Human BM MSCs	Not reported	H5N1	Alveolar epithelial cells	Coculture with MSCs reduces AFC, APP, proinflammatory cytokine responses and prevents down-regulated sodium and chloride transporters [32].
Human UC MSCs	P4-5	H5N1	Alveolar epithelial cells	UC-MSCs correct impaired AFC, APP and restore ion transporters. They also regulate inflammatory responses [34].
Human UC MSCs derived CM	P4-5	H5N1	Alveolar epithelial cells	CM from UC-MSCs restores impaired AFC and APP [34].
Human UC MSCs derived EVs	P4-5	H5N1	Alveolar epithelial cells	UC-MSC exosomes restore impaired AFC and APP [34].
Swine BM MSCs derived EVs	P3-5	H1N1/H7N2/H9N5	Lung epithelial cells	MSC-EVs inhibited influenza virus replication and virus-induced apoptosis in lung epithelial cells [35].
Human BM MSCs	P1-5	Influenza virus	CD8+ T cells	MSCs inhibited proliferation of virus-specificCD8+ T cells and the release of IFN-γ by specific CD8+ T cells [36].

*MSCs* mesenchymal stem/stromal cells, *BM* bone marrow, *UC* umbilical cord, *AFC* alveolar fluid clearance, *EVs* extracellular vesicles, *IFN-γ* interferon γ, *APP* alveolar protein permeability, *CM* conditioned medium

**Table 2:** Stem cell therapy for influenza virus induced lung injury in vivo

Cell sources	Passage number	Influenza virus	Animal models	Other instructions	Biological effect
Human BM MSCs	Not reported	H5N1	Mouse	5×10^5^ cells/mouse injected at 5 dpi	MSCs prevent or reduce virus associated ALI and increase likelihood of survival in the infected mouse [32].
Human UC MSCs	P4-5	H5N1	Mouse	5×10^5^ cells/mouse injected (i.v.) at 5 dpi	UC-MSCs increased the body weight ands lightly improved survival of the infected mice [34].
Mouse BM MSCs	P3-10	H9N2	Mouse	5×10^5^ cells/mouse injected (i.v.) at 30 mpi	MSCs treatment significantly reduces lung injury in mice and is associated with reduced pulmonary inflammation [33].
Swine BM MSCs derived Evs	P3-5	H1N1/H7N2/H9N5	Pig	80μg/kg body weight injected(i.t.)at 12 hpi	MSC-EVs inhibited influenza virus replication and virus induced apoptosis in pig lung epithelial cells [35].
Human/murine BM MSCs	P3/P6-9	H1N1	Mouse	2.5 or 5×10^5^ cells/mouse injected (i.v.) at -2, 0, 2, 5 dpi	MSCs failed to improve survival, decrease pulmonary inflammatory cells or prevent ALI [41].
Human/murine BM MSCs	P7 or less	H1N1	Mouse	5×10^5^ cells/mouse injected (i.v.) at 5/6 dpi	MSCs modestly reduced viral load andfailed to reduce the severity of influenza induced injury [42].
TPR63+/KRT5+ BCs		H1N1	Mouse	The endogenous lung cells	TPR63+/KRT5+ BCs initiate an injury repair process to keep normal lung function by differentiating into mature epithelium [46].
LNEP cells		H1N1	Mouse	The endogenous lung cells	LNEP cells can activate a TPR63+/KRT5+ remodeling program through Notch signaling [48].
KRT5- progenitor cells		H1N1	Mouse	The endogenous lung cells	The SOX2+/SCGB1A-/KRT5- progenitor cells can generate nascent KRT5+ cells [49]. A rare p63+Krt5- progenitor cell population also responds to H1N1 virus-induced severe injury [50].

*MSCs* mesenchymal stem/stromal cells, *BM* bone marrow, *UC* umbilical cord, *EVs* extracellular vesicles, *ALI* acute lung injury, *BCs* basal cells, *LNEPS* lineage-negative epithelial stem/progenitor cells, *i.v.* intravenous, *i.t.* intratracheal, *dpi* days post infection, *mpi* minutes post infection, hpi hours post infection

### Outlook of stem cell therapy for CoV-induced lung injury

Lung injury caused by SARS, MERS, or SARS-CoV-2 poses major clinical management challenges because there is no specific treatment that has been proven to be effective for each infection. Currently, virus- and host-based therapies are the main methods of treatment for spreading CoV infections. Virus- and host-based therapies include monoclonal antibodies and antiviral drugs that target the key proteins and pathways that mediate viral entry and replication [51].The major challenges in the clinical development of novel drugs include a limited number of suitable animal models for SARS-CoV, MERS-CoV, and SARS-CoV-2 infections and the current absence of new SARS and MERS cases [51]. Although the number of cases of SARS-CoV-2-induced pneumonia patients is continuously increasing, antibiotic and antiviral drugs are the primary methods to treat SARS-CoV-2-infected patients. Similar to that of IAV, human CoV-mediated damage to the respiratory epithelium results from both intrinsic viral pathogenicity and a robust host immune response. The excessive immune response contributes to viral clearance and can also worsen the severity of lung injury, including the demise of lung cells [52]. However, the present treatment approaches have a limited effect on lung inflammation and regeneration.

Stem cell therapy for influenza virus-induced lung injury shows promise in preclinical models. Although it is difficult to establish preclinical models of CoV-induced lung injury, we consider stem cell therapies to be effective approaches to improve human CoV-induced lung injury. Acute inflammatory responses are one of the major underlying mechanisms for virus-induced lung injury. Innate immune cells, including neutrophils and inflammatory monocytes-macrophages (IMMs), are major innate leukocyte subsets that protect against viral lung infections [53]. Both neutrophils and IMMs are rapidly recruited to the site of infection and play crucial roles in the host defense against viruses. Neutrophils and IMMs can activate Toll-like receptors (TLRs) and produce interferons (IFNs) and other cytokines/chemokines [54]. There are two functional effects produced by the recruitment of neutrophils and IMMs: the orchestration of effective adaptive T cell responses and the secretion of inflammatory cytokines/chemokines [55]. However, excessive inflammatory cytokine and chemokine secretion impairs antiviral T cell responses, leading to ineffective viral clearance and reduced survival [56].

**Fig. 1:**
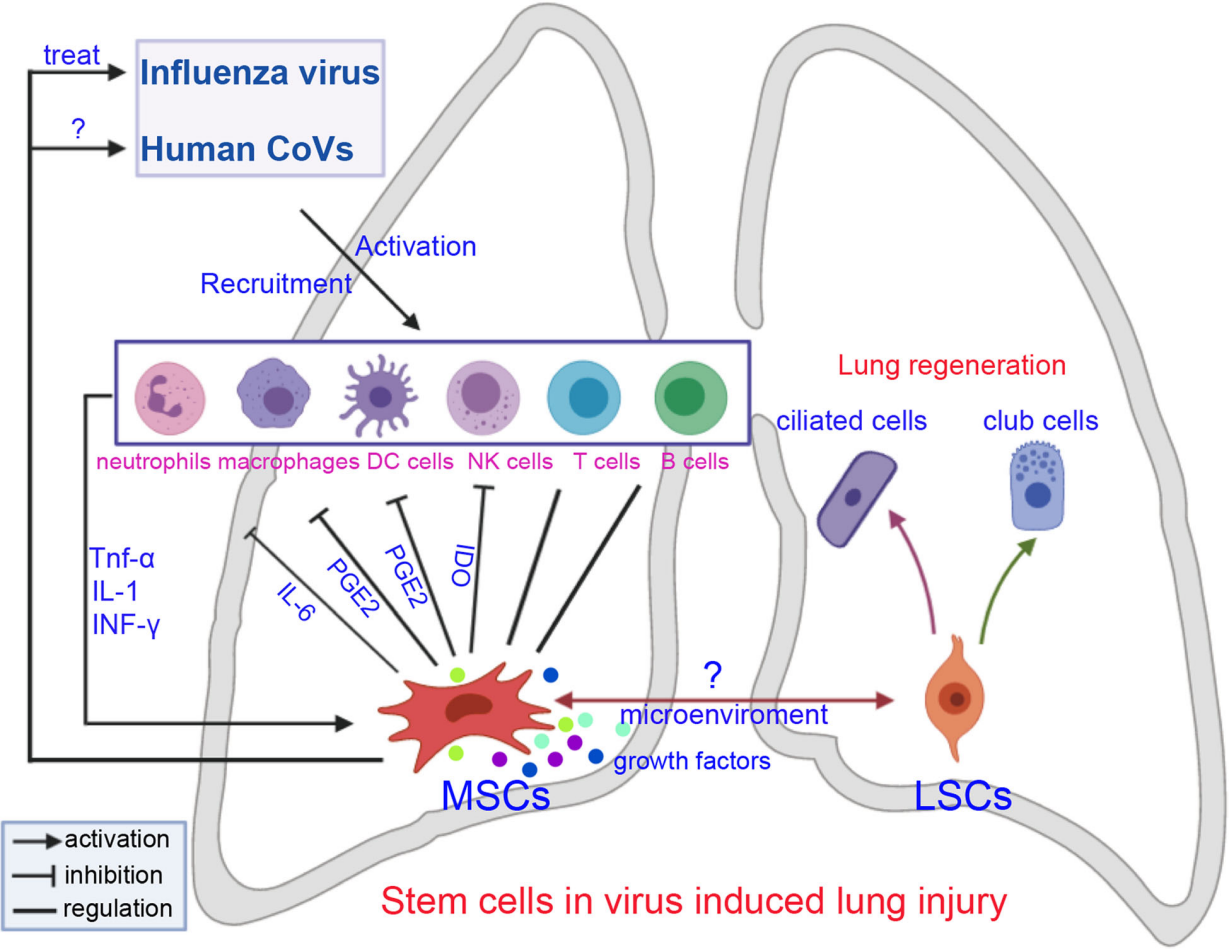
Stem cell therapies for treatment of influenza virus and coronavirus-induced lung injury. CoVs, coronavirus; MSCs, mesenchymal stem/stromal cells; LSCs, lung stem/progenitor cells; NK cells, natural killer cells; DC cells, dendritic cells

MSCs are known to suppress both innate and adaptive immune responses. MSCs have been suggested to inhibit many kinds of immune cells, including T cells, B cells, dendritic cells (DCs), and natural killer (NK) cells in vitro and in vivo [57] (Fig. 1). Several molecules, including IL-1, TNF-α, and INF-γ, most of which are produced by inflammatory cells, are reported to be involved in MSC-mediated immunosuppression [58]. Furthermore, MSCs can produce numerous immunosuppressive molecules, such as IL-6, PGE2, IDO, and IL-10, in response to inflammatory stimuli. PGE2 has been reported to mediate the MSC-mediated suppression of T cells, NK cells, and macrophages. Moreover, PGE2 has been found to act with IDO to alter the proliferation of T cells and NK cells [59]. In contrast, MSCs have come to be recognized as one type of adult stem cell actively participating in tissue repair by closely interacting with inflammatory cells and various other cell types [60]. Numerous reports have demonstrated that MSCs can release an array of growth and inhibitory factors, such as EGF, FGF, PDGF, and VEGF, and express several leukocyte chemokines, such as CXCL9, CCL2, CXCL10, and CXCL11. These factors provide an important microenvironment to activate adaptive immunity for lung repair [61]. Thus, the dual functions of MSCs may improve lung recovery after human CoV-induced ALI. Recently, MSCs was transplanted intravenously to enrolled patients with COVID-19 pneumonia. After treatment, the pulmonary function and symptoms of these patients were significantly improved. Meanwhile, the peripheral lymphocytes were increased, the C-reactive protein decreased, the level of TNF-α was significantly decreased, and the overactivated cytokine-secreting immune cells disappeared. In addition, a group of regulatory DC cell population dramatically increased. Thus, the intravenous transplantation of MSCs was effective for treatment in patients with COVID-19 pneumonia [62, 63].

In addition, endogenous LSCs also play an important role in lung cell reconstitution after virus-induced ALI. In particular, TPR63+/KRT5+ airway BCs comprise approximately equal numbers of stem cells and committed precursors and give rise to differentiated luminal cells during steady state and epithelial repair after lung injury [44, 64]. Research has shown that KRT5+ cells repopulate damaged alveolar parenchyma following influenza virus infection [47]. However, there is still little evidence for the role of altered TPR63+/KRT5+ stem cells during lung injury repair caused by human CoVs.

In summary, exogenous MSCs may modulate human CoV-induced lung injury repair and regeneration through their immunoregulatory properties. These cells are capable of interacting with various types of immune cell, including neutrophils, macrophages, T cells, B cells, NK cells, and DCs. Furthermore, viral infections can activate endogenous LSCs to produce new lung cells and maintain lung function (Fig. 1). Thus, we propose that MSCs and LSCs are two potential cell sources for treating human CoV-induced lung injury.

## Discussion

Despite accumulating evidence on the beneficial effects of MSC administration in preclinical models of influenza virus-induced lung injury, some studies indicate that MSC may not be an effective therapeutic or prophylactic approach to decrease pulmonary inflammation. Darwish and colleagues show MSC therapy fails to improve outcomes in experimental severe influenza [41]. Similar negative results were reported recently by Gotts and colleagues; the researchers find no evidence of beneficial effect of MSCs on weight loss, survival, or lung injury [42]. The timing, dose, route, and frequency of administration of MSCs are significant for the investigation and evaluation of treatment efficiency. Potential reasons for their lack of efficacy include MSCs cannot access to the injured epithelial barrier and MSCs might infect influenza, especially the short duration of preclinical models. The short duration of the murine severe influenza model does not allow for investigation of lung recovery from a process perspective [41, 42]. These experimental studies of MSCs do not preclude the possibility that MSC therapy could potentially contribute to long-term repair and restoration of full lung function following influenza infection.

Besides the exogenous MSCs, tissue-resident MSCs are important regulators of tissue repair or regeneration. Adult pulmonary tissue-resident MSCs demonstrate a phenotype and function similar to BM-MSCs [65]. Currently, few papers reported the status of resident lung MSCs after virus infection-induced lung injury. However, there are some evidence for a role of altered lung MSC function in bleomycin-induced pulmonary arterial hypertension (PAH). In mice, bleomycin treatment induced the loss of these endogenous lung MSCs. Moreover, the resident lung MSCs can regulate the severity of bleomycin-induced injury via modulation of the T cell response [66]. Transplantation of isolated lung MSCs attenuated the bleomycin mediated PAH. In addition, lung MSCs modulated a decrease in numbers of inflammatory cells and inhibition T cell proliferation. These data suggest that lung MSCs function to protect lung integrity following injury; however, when endogenous MSCs are lost, this function is compromised [67]. Although the endogenous lung MSCs reveal similar functions to the exogenous MSCs in non-infectious pneumonia, there is still a lack of relevant evidence to elaborate the relationship between endogenous lung MSCs and human CoV-induced lung injury.

In our opinion, MSCs and LSCs are two potential cell sources for treating influenza virus and human CoV-induced lung injury. Although it is not clear whether MSCs can interact with LSCs, it is a very interesting topic (Fig. 1). Ye and colleagues proved BMSCs affect endogenous lung stem cells (club cells) via cytokines as well as vesicles and activate the Notch signaling thus affecting the proliferation of club cells in phosgene-induced lung injury [68]. Another study shows BMSCs protect against LPS-induced lung injury by restituting alveolar bioenergetics through Cx43-dependent alveolar attachment and mitochondrial transfer [69]. Thus, the transplanted exogenous MSCs may provide an important niche for distinct types of lung cells through different pathways.

The recent emergence of SARS-CoV-2, which is causing an outbreak of unusual viral pneumonia in patients, is another warning of the risks CoVs pose to public health in the world. Although the virus has attacked humans many times, we have few specific approaches to address these virus-induced injuries. We propose that stem cells, including MSCs and LSCs, may be potential methods to treat virus-induced lung injury. Moreover, we have discussed the feasibility and rationality of stem cell therapy from the aspects of immune regulation and lung repair. Although there are still some disadvantages, the clinical trials of stem cell therapy on virus-induced lung injury increased gradually (Table 3). In the future, we expect stem cell therapy to be applied to treat virus-induced lung injury. This review also provides a stem cell therapy strategy for current COVID-19 pandemic.

**Table 3:** Clinic trails of stem cell therapy on influenza virus and CoVs induced lung injury

ID	Cell sources	Virus type	Enrollment	Intervetion	Follow-up	Status	Results	Country
ChiCTR-OCC-15006355	Menstrual blood derived MSCs	H7N9 avian influenza	61 (44/17)	I million cells/kg three times, i.v.	5 years	Completed	MSCs transplantation significantly lowered the mortality	China
NCT02095444	Menstrual blood stem cells	H7N9 avian influenza	20	10^7^ cells/kg, i.v. 4 times in 2 weeks	2.5 years	Unknown	No results posted	China
ChiCTR2000030835	hUC-MSCs	SARS-CoV-2	20	2 or 1 million cells/kg, three times, i.v.	1 year	Recruiting	No results posted	China
ChiCTR2000029990	MSCs	SARS-CoV-2	120 (60/60)	No details	14 months	Recruiting	No results posted	China
NCT04333368	hUC-MSCs	SARS-CoV-2	60	I million cells/kg, three times, i.v.	14 months	Not yet recruiting	No results posted	France
NCT04313322	WJ-MSCs	SARS-CoV-2	5	No details	6 months	Recruiting	No results posted	Jordan
NCT04336254	hDP-MSCs	SARS-CoV-2	20	10^7^ cells/kg, i.v. 3 times in 1 week	1 year	Recruiting	No results posted	China

All information was extracted from http://www.chictr.org.cn/ and https://www.clinicaltrials.gov/

*MSCs* mesenchymal stem/stromal cells, *WJ* Wharton’s jelly, *i.v.* intravenous, *DP* dental pulp, *UC* umbilical cord

## Data Availability

Not applicable.

## Abbreviations

ALI: Acute lung injury
ARDS: Acute respiratory distress syndrome
CoVs: Coronavirus
CMVs: Cytomegalovirus
RSVs: Respiratory syncytial virus
IAV: Influenza A virus
HA: Hemagglutinin
NA: Neuraminidase
SARS-CoV: Severe acute respiratory syndrome coronavirus
MERS-CoV: Middle east respiratory syndrome coronavirus
MSCs: Mesenchymal stem/stromal cells
AFC: Alveolar fluid clearance
APP: Alveolar protein permeability
Ang1: Angiopoietin-1
KGF: Keratinocyte growth factor
FGF2: Fibroblast growth factor 2
EVs: Extracellular vesicles
BCs: Basal cells
LNEPs: Lineage-negative epithelial stem/progenitor cells
LSCs: Lung stem/progenitor cells
IMMs: Inflammatory monocytes-macrophages
TLRs: Toll-like receptors
IFNs: Interferons
NK: Natural killer
COVID-19: Coronavirus disease 2019
